# Antigen distribution of TMUV and GPV are coincident with the expression profiles of CD8α-positive cells and goose IFNγ

**DOI:** 10.1038/srep25545

**Published:** 2016-05-06

**Authors:** Hao Zhou, Shun Chen, Mingshu Wang, Renyong Jia, Dekang Zhu, Mafeng Liu, Fei Liu, Qiao Yang, Ying Wu, Kunfeng Sun, Xiaoyue Chen, Bo Jing, Anchun Cheng

**Affiliations:** 1 Institute of Preventive Veterinary Medicine, Sichuan Agricultural University, Chengdu, Sichuan 611130, China; 2 Avian Disease Research Center, College of Veterinary Medicine of Sichuan Agricultural University, Chengdu, Sichuan 611130, China; 3 Key Laboratory of Animal Disease and Human Health of Sichuan Province, Sichuan Agricultural University, Chengdu, Sichuan 611130, China

## Abstract

Both Tembusu virus (TMUV) and goose parvovirus (GPV) are causative agents of goose disease. However, the host immune response of the goose against these two different categories of virus has not been well documented. Here, we compared the clinical symptoms and pathological characteristics, antigen distribution and intensity, and expression of immune-related genes in TMUV- and GPV- infected goose. The immunohistochemistry analysis demonstrated that GPV was primarily located in the liver, lung, small intestine, and rectum, while TMUV was situated in the liver, brain, spleen, and small intestine. The induction of IFNγ and proinflammatory cytokines is highly associated with the distribution profiles of antigen and CD8α+ molecules. The effector function of CD8 T cells may be accomplished by the secretion of IFNγ together with high expression of proinflammatory cytokines such as IL1 and IL6. Remarkably, significant increases in the transcription of immune genes were observed after infection, which suggested that both GPV and TMUV can effectively induce immune response in goose PMBCs. This study will provide fundamental information for goose molecular immunology in defending against pandemic viruses.

Since 2010, the Tembusu virus (TMUV) has caused significant economic losses in the poultry industry in China^1^,^2^,^3^, including reduced egg production, especially in duck farms. Considering its similarities with flavivirus, it may also pose a potential threat to public health^4^. It is a single-stranded RNA virus and a member of the Flavivirus genus in the family Flaviviridae, with three structural proteins including the core (C) protein, pre-membrane (prM) protein, envelope (E) protein, and seven nonstructural (NS) proteins (NS1, NS2A, NS2B, NS3, NS4A, NS4B, NS5). TMUV has been routinely isolated from a wide range of host species including mosquitos, chickens, ducks, geese, pigeons, sparrows, and the evolutionary factors have been identified^5^. It has been found that the TMUV strain (PTD2010) is detectable in most adult duck organs such as the brain, spleen, ovary and intestinal tract after inoculation^6^. Chickens, ducks and geese are all susceptible to TMUV^2^,^7^,^8^. Recent studies revealed that age was correlated with the pathogenesis of TMUV, as juvenile ducks were more susceptible to TMUV than older ducks^9^. The expression of immune-related genes was systematically examined in ducks after TMUV infection^10^. Aquatic birds, especially geese, are regarded as the reservoir for many pandemic viruses such as the avian influenza virus. However, until now, little information has been available about TMUV immunological activities, pathogenesis and viral antigen distribution in geese.

Additionally, parvoviruses, a type of DNA virus with a single strand of DNA wrapped in an icosahedral capsid, can be found widely in a host of species, including birds and mammals. Goose parvovirus (GPV), a member of the parvovirus family and causative agent of Derzsy’s disease in goslings and Muscovy ducklings, has a genome of approximately 5 k nucleotides long, containing large left and right open reading frames that encode the two nonstructural proteins (NS1, NS2) and three capsid proteins (VP1, VP2, and VP3)^11^,^12^,^13^. GPV, with relatively high mortality and morbidity of goslings, has become widespread in most goose farming countries, resulting in a detrimental effect on the poultry industry. Research also showed significant genetic recombination between GPV and Muscovy Duck parvovirus (MDPV)^14^,^15^,^16^, indicating that GPV is currently involved in rapid and intriguing evolution in the hosts to enhance its adaption. However, information about the molecular mechanism and immunological activity of the goose in defending against viral infection remains poorly understood.

There is a paucity of information available on whether the goose plays a distinct and special role in the immune defence and regulatory defence against these viruses. It is vital to identify and characterize the goose immune response against TMUV and GPV, as representative pathogenic RNA and DNA viruses, respectively. Moreover, the ecological characteristics and pathogenesis of TMUV and GPV in the goose are currently inadequately defined. To explore the similarity and particularity of the goose immune response against TMUV and GPV and to clarify the antigen distribution, histopathology, and its immune effects in infected geese, here, artificial infection of TMUV and GPV in geese was performed, followed by the immunohistochemical detection of antigens and analysis in immune-related and non-immune tissues of infected geese. The goose antiviral related immune response (type I interferon (IFNα), type II interferon (IFNγ), IL1β and IL6) was determined. Furthermore, a goose peripheral blood mononuclear cells (PBMCs) model was chosen for challenge by agonist and viruses *in vitro*, which may be essential in the development of vaccine adjuvants or novel strategies to defend against viral infection. Accordingly, the comparison between these two different types of virus has been comprehensively investigated.

## Results

### Clinical syndromes and histopathological assay

Both the GPV- and TMUV-infected groups showed depression and lassitude, inability to stand steadily and loss of appetite. TMUV-infected animals exhibited syndromes of depression, reluctance to move, ruffled hair, diarrhoea, marked depression, severe diarrhoea and paralysis by 5 dpi compared with the absence of clinical symptoms or necrotic lesions in the control group. One death occurred suddenly at 5 dpi. Additionally, the average body weight of the GPV group and the TMUV group both decreased significantly compared to the control group (P < 0.01; P < 0.01) (Supplementary Fig. S1). At 5 dpi, distinct clinical syndromes were observed in geese infected with the different viruses infected goose. There were manifest superficial syndromes in the TMUV-infected group, with mesenterium, liver swelling, marginal necrosis, hyperaemia cerebri and meninges. The loss of ruffled feathers was clearly observed on the backsides of GPV-infected geese. Extreme weakness, inappetence, paralysis, ataxia, convulsion, and opisthotonos were observed in the TMUV-infected geese. Significantly, the weight of the geese showed an obvious decreasing trend after infection. Pathological results (HE staining) showed the congestion and haemorrhage of the small mesenteric bowel in TMUV-infected geese. Meanwhile, meningeal congestion and encephalomalacia in the brain, splenomegaly in the spleen, crimson hepatomegaly, congestion and haemorrhage in the liver were observed (Supplementary Fig. S2).

### Immunohistochemical staining

**Figure 1:**
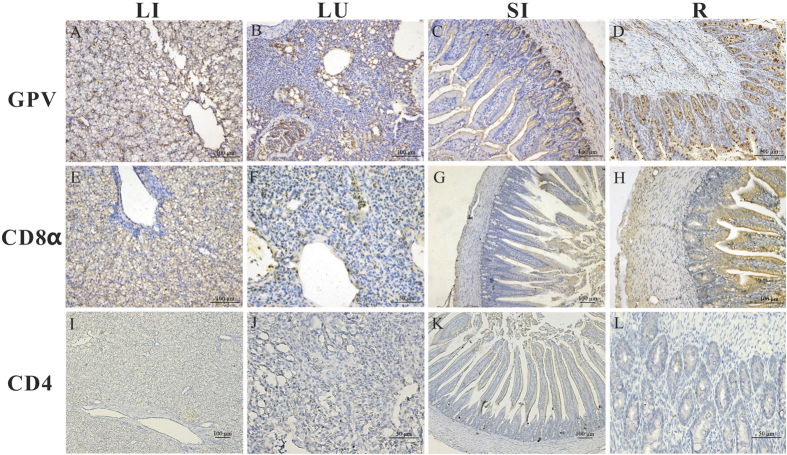
The location and density of GPV antigen, CD4 and CD8α molecules in the liver (LI), lungs (LU), small intestine (SI), and rectum (R). Geese were humanly killed 5 days post infection by viruses. The protein locations in the different tissues of H9N2-infected birds were detected by IHC assay. Positive virus signals were detected, cells positive for CD4 or CD8α antigen appeared dark brown using immunohistochemical staining, and sections were counterstained with haematoxylin. Mouse polyclonal antibody against GPV was prepared by our laboratory. The dilution folds of mouse anti-duck monoclonal CD4 antibodies (AbD Serotec MCA2478) and mouse anti-goose monoclonal CD8α (provided by our laboratory) antibodies were 1:100, respectively. Incubation with goat anti-mouse or goat anti-rabbit secondary antibody was performed according to the protocols of the immunoassay kit. Liver (**A**,**E**,**I**), lungs (**B**,**F**,**J**), small intestine (**C**,**G**,**K**) and rectum (**D**,**H**,**L**).

To explore the antigen location in different tissues during infection, we primarily determined the viruses, CD4+ cell and CD8α+ cell locations by IHC staining assays. For the GPV infection group (5 dpi), the GPV antigen was predominantly and strongly distributed in the liver, lungs, small intestine, and rectum (+++), moderately detected in the spleen (++), and faintly detected in the brain (+) (Fig. 1A–D, Table 1). Importantly, the antigen was readily and widely distributed in the mucous membrane epithelium and myofibrocytes of the intestines (Fig. 1C–D). Interestingly, CD8α cells were widely scattered through the liver and lungs (+++) (Fig. 1E,F) and most prevalent in the mucous membrane epithelium and myofibrocytes of the rectum (+++) (Fig. 1H). CD4 positive cells were weakly and faintly detected (Table 1).

**Figure 2:**
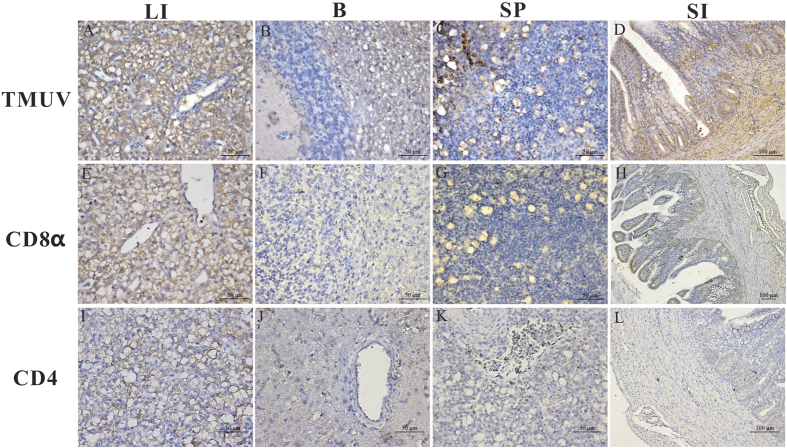
The location and density of TMUV antigen, CD4 and CD8α molecule in the liver (LI), brain (B), spleen (SP), and small intestine (SI). Geese were humanly killed 5 days post infection by viruses. These protein locations in the different tissues of H9N2-infected birds were detected by IHC assay. Positive virus signals were detected, cells positive for CD4 or CD8α antigen appeared dark brown using immunohistochemical staining, and sections were counterstained with haematoxylin. Rabbit polyclonal antibody against TUMV E protein was prepared by our laboratory. The dilution folds of mouse anti-duck monoclonal CD4 antibodies and mouse anti-goose monoclonal CD8α antibodies were both 1:100. Incubation of goat anti-mouse or goat anti-rabbit secondary antibody was performed by the protocols of the immunoassay kit. Liver (**A**,**E**,**I**), brain (**B**,**F**,**J**), spleen (**C**,**G**,**K**) and small intestine (**D**,**H**,**L**).

For the TMUV infection group (5 dpi), the TMUV antigen was predominantly and strongly distributed in the liver, brain, spleen, and small intestine (+++), and moderately detected in the lungs (++)(Fig. 2A–D, Table 1). Importantly, the antigen was readily and widely distributed in the mucous membrane epithelium and myofibrocytes of the intestines (Fig. 2D). CD8α-positive cells were mainly scattered through the liver and spleen (++) (Fig. 2E,G) and most prevalent in the medulla and epithelial reticular cells of the small intestine (++) (Fig. 2H) but relatively faintly detected in the lungs (++) and heart (+) (Table 1). Furthermore, CD4-positive cells could be detected in the hepatocytes and mesenchymes of the liver and were weakly and faintly detected in the spleen, lungs, and rectum (+) (Fig. 2I–L).

### Antiviral cellular immune response against infection *in vivo* and *in vitro*

**Figure 3:**
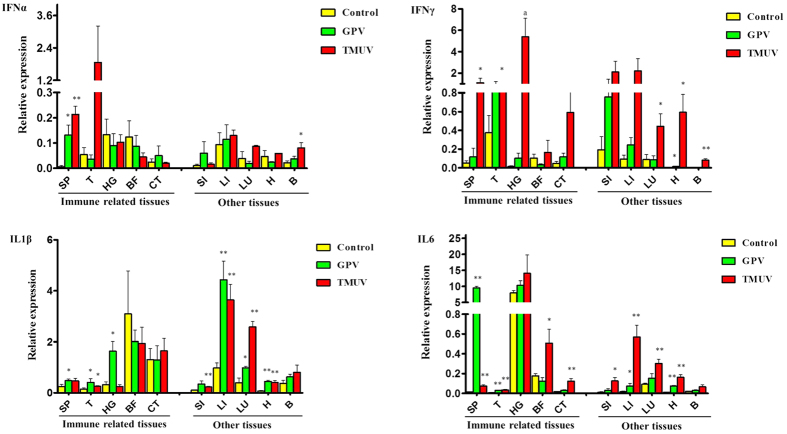
Comparative analysis of goose immune-related genes including IFNα, IFNγ, IL1β, and IL6 in GPV- and TMUV-infected goose tissues. Geese were humanly killed 5 days post infection by viruses. These gene transcripts in goose tissues were amplified by real-time qPCR; β actin was amplified as an internal control. HG: Harderian gland, BF: bursa of fabricius, T: thymus, CT: caecal tonsils, SP: spleen, SI: small intestine, LI: liver, LU: lung, H: heart, TR: trachea. Groups marked ‘*’ presented a significant difference at P < 0.05, and groups marked ‘**’ represented a significant difference at P < 0.01. ‘ns’, not significant. The data is the representative of three independent experiments.

*In vivo*, IFNα and IFNγ were not upregulated in the brain after GPV infection (Fig. 3), although interferon genes were differentially regulated in all tested tissues but showed an upward trend, and the IFNγ response was fairly strong after infection. Significant upregulation of IFNα expression was detected in the spleen during GPV and TMUV infection, and interestingly, significant upregulation of IFNα expression was shown in the brain of TMUV-infected birds.

Meanwhile, significant upregulation of IFNγ expression was detected in almost all TMUV-infected samples, also including the brain. Goose IL1β expression in the GPV-infected group increased both in immune-related tissues such as the spleen (P < 0.05), thymus (P < 0.05), and Harderian gland (P < 0.05) and in other tissues including the liver (P < 0.01), lungs (P < 0.05) and heart (P < 0.01) (Fig. 3). Approximately 2 to 5-fold more IL1β gene transcript was observed in the spleen, liver, lungs, and brain of TMUV-infected animals compared to mock-infected geese at 5 dpi. More than 5-fold IL6 gene transcript was observed in the liver, spleen, and heart of GPV-infected geese, depending on the individual animal. In the TMUV-infected group, the goose IL6 expression increased in almost all tissues, with significant increases in the spleen (P < 0.01), thymus (P < 0.01), bursa of fabricius (P < 0.05), and caecal tonsils (P < 0.01). This upward trend was also observed in other tissues such as the liver and lungs. Although the goose IL6 gene was significantly highly expressed in almost all tissues of TMUV-infected birds, it was moderately expressed in the brain and minimally expressed in the thymus.

**Figure 4:**
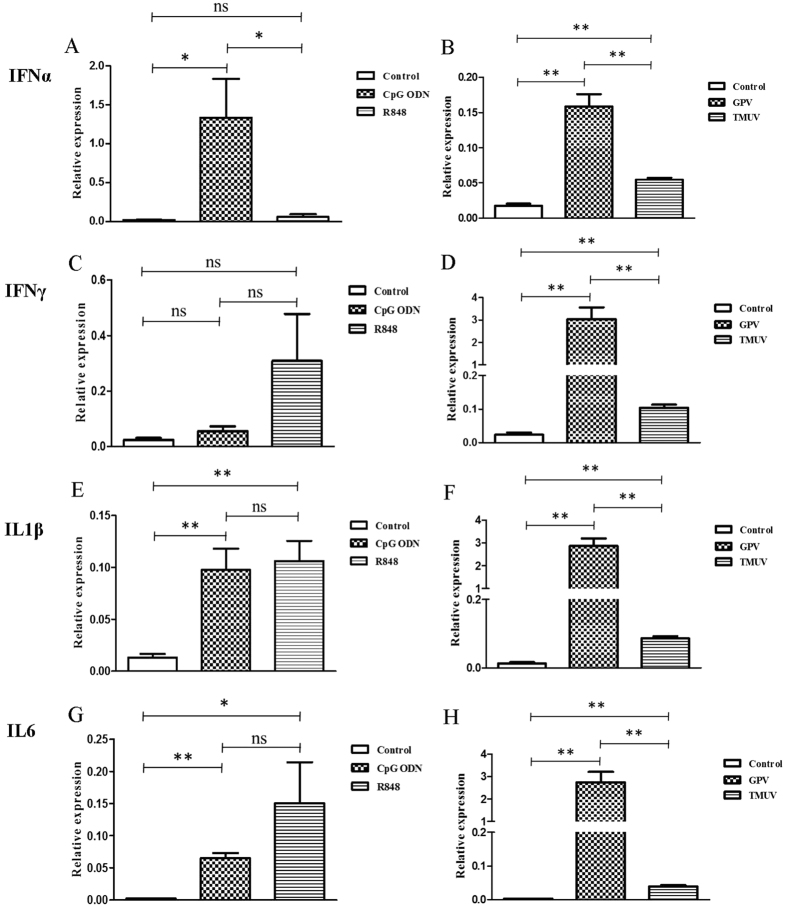
Relative transcriptional levels in goose PBMCs at 6 hours after stimulation with R848, CpGODN, GPV and TMUV. IFNα, IFNγ, IL1β, and IL6 mRNA expression levels were normalized to internal control. Data indicated are expressed as the mean ± SEM (n = 4). Groups marked by one star (*) presented a significant difference at P < 0.05; groups marked by two stars (**) presented a significant difference at P < 0.01. The data is the representative of three independent experiments.

*In vitro*, at 6 hours post stimulation, IFNα was more regulated by CpG ODN (P < 0.05) (Fig. 4A), while IFNγ was more stimulated by R848 in goose PBMCs. IL1β was significantly up-regulated in the goose PBMCs following treatment with R848 (P < 0.01) or CpG ODN (P < 0.01) (Fig. 4E). IL6 also showed a substantial upward trend in transcriptional level in response to R848 (P < 0.05) and CpG ODN (P < 0.01) (Fig. 4G). However, significant increases in the transcription of goose IFNα, IFNγ, IL1β, and IL6 were all observed after infection with these two types of virus (TMUV and GPV) (P < 0.01; P < 0.01; P < 0.01; P < 0.01) (Fig. 4B,D,F,H). Goose IFNα have been observed to have a significant difference between CpG ODN and R848 group (Fig. 4A). The significant differences of these cytokine expression between TMUV and GPV group have been observed (P < 0.01; P < 0.01; P < 0.01; P < 0.01) (Fig. 4B,D,F,H).

## Discussion

The immune system is an effective defence to protect hosts from viral infection. When host cells encounter pathogens, antigen processing cells (APCs) capture and transfer the foreign antigen to T lymphocytes with the expression of MHC-I and MHC-II molecules^17^, which can be further differentiated into CD4-positive T cells and CD8-positive T cells^18^. The activated CD4+ T cells produce various cytokines, such as IFNγ, to further promote the antibody production and macrophages to phagocytose microbes^19^, while CD8+ T cells kill specific cells infected with intracellular antigens with cytotoxic granules^20^. As one of two lymphocyte types, B lymphocytes can capture foreign pathogens without the help of APCs, produce antibody to destroy the foreign antigen^21^ and release a broad variety of cytokines^22^. However, pathogen-associated molecular patterns of viral antigens were also recognized by pattern recognition receptors (PRRs) in birds^23^, located in the endosome, plasma or cellular membrane (e.g., TLRs, RIG-I, MDA5, LGP2). The cytokines, including proinflammatory cytokines and interferon, were subsequently produced to clear the viruses. Although an important natural reservoir of avian virus, little information is available on the molecular mechanisms of innate antiviral immunity in the goose. Here, two causative agents of goose disease, Tembusu virus (TMUV) and goose parvovirus (GPV), were chosen. The tissue distribution of CD4-positive and CD8-positive cells, GPV and TMUV location, and the expression levels of IFNs and proinflammatory cytokines *in vitro* and *in vivo* were primarily explored.

In this study, a series of clinical signs of TMUV-infected geese at 5 dpi were shown, such as acute anorexia and neurological disorders. Pathological changes, including enlarged liver with haemorrhage, congestive meninx, and small intestine mucosal swelling and haemorrhage, were compatible with the high pervasive virulence of TMUV, similar to the histopathological changes in duck^9^. Geese infected by the duck TMUV strain can exhibit neurological dysfunction, which is consistent with the neurological syndromes of ataxia and paralysis in ducklings^24^ and neurovirulence in intracerebrally inoculated BALB/c mice^25^. Regarding GPV, little clinical syndrome was observed except for a loss of tidy feathers. One hypothesis regarding this asymptomatic phenomenon of viral infection in geese is that GPV replication is likely to be controlled by the innate immune response. Additionally, as noted in the current data, TMUV infection seems to be more virulent than GPV infection. However, as yet there has been no convincing definition of the differences in immune response induced by the two viruses.

In most of the tissues investigated in this study, a positive signal was found in the sinus endothelial cells of the liver. The GPV strain was also found to be distributed in the upper respiratory system, such as the lungs, and the intestinal digestive system, such as the small intestine and rectum. These results is consistent with the GPV-caused disease characterized by enteritis and hepatitis in different regions^26^,^27^.Positive antigen signal in the spleen was observed in GPV-infected goslings, indicting the important role of the cellular immune response against GPV infection. Consistent with the reference, in experimentally GPV-infected goslings, cells in the spleen and bone marrow were discovered to be the targets for GPV infection^28^. TMUV has been tested in the brains of different aged ducks at 3 or 5 days post infection^9^. In ducks infected with TMUV at 5 days of age, heavily IHC-stained cells were mostly observed among glial cells in the brain and splenic cells^29^. Similarly, a positive signal of TMUV was found in the spleen and brain, with stronger signals particularly in the liver and small intestine, implying that TMUV might invade the immune organs and induce a strong cytokine storm, ultimately having a detrimental impact on the goose nervous system.

The activated cytotoxic T lymphocytes play a pivotal role in virus clearance. In this study, high expression patterns of goose CD8α were observed in the intestinal tracts and liver, where the antigens are primarily located (both TMUV and GPV). This is consistent with the previous reports that goose CD8+ T cells in spleen mononuclear cells substantially increased from 72 h after GPV infection^30^. Interestingly, both TMUV-infected and CD8α+ positive cells are widely scattered in the brain during infection. Overall, the CD8α protein distribution preference and viral antigen location showed similar distributions, which suggested an immunoregulatory function in infection defence. As reported previously, CD8+ T cells kill the infected cells via the release of cytotoxic granules such as perforin and granzymes^20^, while the CD4+ T cells influence the antiviral process and humoral immunity by secreting immune-related cytokines such as IFNγ^19^. Herein, both the CD8+ and CD4+ T cells were involved in an IFN-mediated antiviral immune response against viral infection. As the canonical Th1 cytokine, IFNγ is vital for innate and adaptive immunity against viral infection. IFNγ was highly produced by the virus-specific CD8+ T cells by immune regulation^31^. This behaviour partly contributes to the fact that the induction effect on IFNγ proliferation was consistent with the activated CD8+ T cells. IFNγ, in turn, increases the expression of MHC class I molecules to make infected cells more easily and readily recognizable by CD8+ cytotoxic T cells. Thus, IFNγ can be induced by CD8+ T cells and is also a helper to strengthen CD8+ cytotoxic T cells. Moreover, IFNγ can activate macrophages to kill microbes and other related cells to perform distinct functions as well as to induce the apoptosis of epithelial cells^32^. Based on our data, the up-regulation of goose IFNγ mRNA levels was detected in the thymus, intestine, and liver of GPV-infected birds and in almost all tissues of TMUV-infected birds. Unexpectedly, the high IFNγ expression tissues were the main pathogen-invaded tissues, as well as the tissues where CD8α positive cells were distributed.

Collectively, goose IFNγ produced by GPV- and TMUV-challenged T cells (mostly CD4+ cells) can promote CD8+ cytotoxic T cells, further activating B cells to produce antibodies, as well as activating phagocytes to kill microbes, which play an important role in the goose host anti-GPV and anti-TMUV immune response. IL1β is a major potent proinflammatory cytokine with pleiotropic effects on the immune system, which is widely produced in goose organs during GPV and TMUV infection. It has been reported that IL1β is widely involved in various cellular activities, including the induction of proinflammatory proteins, haematopoiesis, and cell differentiation^32^. IL6 has been considerably induced in the spleen of the GPV-infected group, and its expression level rose clearly both in immune-related and non-immune tissues during TMUV infection, most likely due to the stronger virulence of the TMUV strain. IL6 is a pleiotropic cytokine that contributes to antiviral immunity and could regulate the development and differentiation of immune-related cells^33^ and the development of T helper 17 cells^34^, as well as fostering B cell development and IgG antibody production^33^,^35^,^36^. It has also been suggested that the IL6 signalling pathway induces the promotion of CD8+ T cell^37^. High IL6 expression was observed in the goose liver in the virally infected group, together with previous reports that naive CD8+ T cells are also stimulated in peripheral organs such as the liver^38^, which suggested that proinflammatory cytokines influence CD8 T cell activation. It is suggested that the ensemble of CD8-positive signals in tissues was associated with the increased level of proinflammatory cytokines (IL1β and IL6) during both GPV and TMUV infection.

Viral-associated molecular pattern recognition by the host innate immune response relies on Pattern Recognition Receptors (PRRs), which are vital for defending the host from invading pathogens. The ligands for TLR3 are dsRNA derived from viruses, while the ssRNA for TLR7 originated from RNA viruses. After recognition, downstream signal transduction (NF-κв or IRF3/7) was activated, ultimately inducing the production of interferons and inflammatory cytokines^39^,^40^,^41^. In the innate immune response, interferon is an effective cytokine that orchestrates various types of distinct cellular antiviral immune responses through inducing large numbers of interferon-stimulated genes, which have a direct and dramatic antiviral function. TMUV is a single = stranded RNA virus, while GPV is a single-stranded DNA virus. With our *in vivo* data in mind, we try to confirm our assumption by *in vitro* study. The stimulation of goose PBMCs with R848 (TLR7 agonist) or CpG-ODN (TLR21 agonist) can produce a strong immune response, including the upregulation of interferons (IFNα and IFNγ) and the significant upregulation of proinflammatory cytokines (IL1β and IL6) (P < 0.05). As expected, both the interferons (IFNα and IFNγ) and the proinflammatory cytokines (IL1β and IL6) were extremely significantly upregulated by GPV and TMUV *in vitro* (P < 0.01). These results reveal that the two signal transduction pathways show partly similar downstream cytokines through the IRF3/7 and NF-κв mediated pathway. Comparing the DNA virus and RNA virus, the ssRNA virus is supposed to be sensed by endosome TLR7, while DNA is supposed to be sensed by TLR21 or other PRR sensors. Importantly, the activation of specific TLRs can strongly promote the antiviral cellular immune function. The immune potency of GPV was stronger than the immune potency of TMUV according to *in vitro* studies, which is different from the results *in vivo*. The substantial differences *in vitro* and *in vivo* demonstrate the complicated immune regulation in geese. The antiviral signal pathway of the RNA virus and DNA virus should be explored further to elucidate this identity. Whether this investigation can be achieved remains to be determined, but if successful, it would represent a significant advance in our understanding of antiviral immunity and our potential to develop effective antiviral immunotherapies for pandemic viruses.

## Conclusion

In this report, the TMUV and GPV antigen distribution and goose antiviral immune response induced by infection have been elucidated for the first time. The expression tendencies of goose immune-related genes after artificial infection by viruses were distinctly upregulated. The antigen distribution of TMUV and GPV during infection in geese was coincident with the CD8+ T cell distribution, which was also associated with the high production of IFNγ and proinflammatory cytokine (IL1β and IL6) in virus-preferable tissues. It is suggested that goose IFNγ produced by GPV- and TMUV-challenged T cells (mostly CD4+ cells) can promote CD8+ cytotoxic T cells, further activate B cells to produce antibody, and activate phagocytes to kill microbes, thus playing an important role in the goose host anti-GPV and anti-TMUV immune response. The functions of CD8 T cells may be associated with the secretion of IFNγ together with the higher expression of proinflammatory cytokines such as IL1 and IL6. TMUV was observed to have the stronger potential to stimulate the goose immune and inflammatory response. This accumulating evidence sheds new light on the crucial role played by goose interferons and proinflammatory cytokines, which is critical in understanding the underlying mechanism of the innate response of geese in the defence against TMUV and GPV.

## Materials and Methods

### Animal ethics

The animal studies were approved by the Institutional Animal Care and Use Committee of Sichuan Agricultural University and followed the National Institutes of Health guidelines for the performance of animal experiments. The viral challenge in this study was conducted on 3-day-old geese.

### Virus and reagents

GPV and TUMV viruses were kindly provided by the department of Preventive Veterinary Medicine, Sichuan Agricultural University. The previously reported egg infectious dose was 10^−6.6^ EID_50_/0.2 ml for GPV^30^, while the infectious titre of this viral strain of TMUV was measured as 6.3 × 10^6^ TCID_50_/ml, as previously described^42^. R848 (InvivoGen, San Diego, CA) (250 μg/ml) and CpG ODN 2006 (Sangon Biotech, China) (1 mg/ml) were prepared before the experiments.

Mouse polyclonal antibody against GPV and rabbit polyclonal antibody against E protein of TUMV was prepared by our laboratory. Mouse anti-duck monoclonal CD4 antibodies (AbD Serotec MCA2478) and mouse anti-goose monoclonal CD8α (provided by our laboratory) antibodies were used. Goat anti-mouse and goat anti-rabbit antibody were purchased from ZSGB-BIO, Beijing, China. All the steps were conducted according to the protocols of the immune assay kit (ZSGB-BIO, Beijing, China).

### *In vivo* study

The GPV-infected group was inoculated using intramuscular and oral administration (0.5 ml), while the TUMV group was infected by subcutaneous injection (0.5 ml). The control group was inoculated with 0.5 ml sterile phosphate buffered saline (PBS) in the same manner as the TMUV group. These geese were all sacrificed at 5 days post infection (5 dpi). Various tissues including the thymus (T), spleen (SP), bursa of fabricius (BF), caecal tonsils (CT), Harderian gland (HG), small intestine (SI), brain (B), pancreas (P), rectum (R), heart (H), lung (LU), trachea (TR), and liver (LI) were isolated. Total RNA was isolated from selected tissues using the RNAiso Plus reagent (Takara Bio, Otsu, Japan). Complementary DNA was synthesized from 2 μg of total RNA using a 5X All-In-One RT MasterMix Reagent Kit (Applied Biological Materials, Richmond, BC, Canada). Specific primers of genes used in real-time PCR (RT-PCR) are listed in Table 2. The cycle threshold value was normalized to the endogenous housekeeping gene β-actin. Goose IFNα, IFNγ, IL-1β, and IL-6 were determined by the 2^−ΔΔCT^ method by RT-PCR conducted in the Bio-Rad system, as previously described^43^.

### *In vitro* study

Goose PBMCs were separated by Ficoll gradient (TBD, Tianjin, China) according to the protocols. The PBMCs were grown overnight in RPMI 1640 supplemented with 10% foetal bovine serum (Gibco, Carlsbad, CA, USA) at 37 °C, 5% CO_2_ with a cell density of approximately 1.0 × 10^7^ cells/well in 6-well plates. After 6 hours of stable incubation, the indicated stimulants were added to the wells. The working concentration of stimulants was 5 μg/ml for R848, 10 μg/ml for CpG-ODN, 50 μl for TMUV and 50 μl for GPV. After that, 6 h time points were taken in 3 identical wells. Cells incubated with PBS served as the controls. Then, total RNA was isolated from cells and reverse transcribed into cDNA as described above and stored at −80 °C for further use. The expression of interferons (IFNα and IFNγ) and inflammatory cytokines (IL-1β and IL-6) in the samples were detected by RT-PCR as described above.

### Immunohistochemical analysis

IHC staining with sight modification was performed according to previously described protocols^44^. Mouse polyclonal antibody against GPV was in diluted 1:100, and rabbit polyclonal antibody against E protein of TUMV was diluted 1:800, as the primary antibodies. After incubation with the primary antibody overnight at 4 °C followed by washing three times with PBST, the sections were incubated with goat anti-mouse or goat anti-rabbit secondary antibody (Biotin-Streptavidin HRP Detection Systems, ZSGB-BIO, Beijing, China) for 30 minutes at 37 °C. All procedures were performed according to the protocols of the immunoassay kit. The primary antibodies for GPV and TMUV were 1:100 and 1:800, respectively, and both the CD4 and CD8α primary antibodies were fold diluted to 1:100. The intensity of immunoreactivity was subjectively scored using the following system, according to the references^44^: no detectable antigen (−); weak, antigen faintly detected (+); moderate, antigen readily detected (++); strong, antigen staining intense (+++). Histopathology samples of goose tissues were routinely dehydrated, embedded in paraffin and stained with haematoxylin and eosin (HE), then observed under a light microscope.

### Statistical analysis

The RT-qPCR data were analyzed by the 2^−ΔΔCT^ method using Bio-Rad CFX Manager Software. Data are indicated as the mean and standard deviation. Significant differences between the two groups were determined using Student’s t test. P values less than 0.05 were considered to represent statistically significant differences.

## Additional Information

**How to cite this article**: Zhou, H. *et al*. Antigen distribution of TMUV and GPV are coincident with the expression profiles of CD8a-positive cells and goose IFNγ. *Sci. Rep*. **6**, 25545; doi: 10.1038/srep25545 (2016).

## Supplementary Material

Supplementary Information

## Figures and Tables

**Table 1:** The intensity of immunoreactivity (GPV, TMUV, CD8α, and CD4) was scored subjectively.

Tissue name	Brain	Lung	Liver	Small intestine	Rectum	Spleen	Heart
GPV	+	+++	+++	+++	+++	++	–
CD8α	++	+++	+++	+++	+++	++	–
CD4	+	+	+	+	+	+	–
TMUV	+++	++	+++	+++	+++	++	++
CD8α	+	++	++	++	+++	++	+
CD4	+	+	+	+	+	+	–

The birds were humanly killed at 5 days post infection, and the selected tissues were isolated for IHC analysis. No detectable antigen (−); weak, antigen faintly detected (+); moderate, antigen readily detected (++); strong, antigen staining intense (+++).

**Table 2:** List of primers used in this study and their sequences.

Primers name	Sequences
IFNα F	CAGCACCACATCCACCAC
IFNα R	TACTTGTTGATGCCGAGGT
IFNγ F	TGAGCCAGATTGTTTCCC
IFNγ R	CAGGTCCACGAGGTCTTT
IL1β F	TCCGCCAGCCGCAAAGTG
IL1β R	CGCTCATCACGCAGGACA
IL6 F	AAGTTGAGTCGCTGTGCT
IL6 R	GCTTTGTGAGGAGGGATT
βactin F	CCGTGACATCAAGGAGAA
βactin R	GAAGGATGGCTGGAAGAG
