# Corrigendum: Antigen distribution of TMUV and GPV are coincident with the expression profiles of CD8α-positive cells and goose IFNγ

**DOI:** 10.1038/srep44518

**Published:** 2017-03-28

**Authors:** Hao Zhou, Shun Chen, Mingshu Wang, Renyong Jia, Dekang Zhu, Mafeng Liu, Fei Liu, Qiao Yang, Ying Wu, Kunfeng Sun, Xiaoyue Chen, Bo Jing, Anchun Cheng

Scientific Reports 6: Article number: 2554510.1038/srep25545; published online: 05 06 2016; updated: 03 28 2017

This Article contains errors in the Figure legends of Figure 1 and Figure 2. The correct Figure legends appear below

**Figure 1. The location and density of GPV antigen, CD4 and CD8α molecules in the liver (LI), lung (LU), small intestine (SI), and rectum (R).** Geese were humanly killed 5 days post infection by viruses. The protein locations in the different tissues of GPV-infected birds were detected by IHC assay. Positive virus signals were detected, cells positive for CD4 or CD8α antigen appeared dark brown using immunohistochemical staining, and sections were counterstained with haematoxylin. Mouse polyclonal antibody against GPV was prepared by our laboratory. The dilution folds of mouse anti-duck monoclonal CD4 antibodies (AbD Serotec MCA2478) and mouse anti-goose monoclonal CD8α (provided by our laboratory) antibodies were 1:100, respectively. Incubation with goat anti-mouse or goat anti-rabbit secondary antibody was performed according to the protocols of the immunoassay kit. Liver (**A**,**E**,**I**), lung (**B**,**F**,**J**), small intestine (**C**,**G**,**K**) and rectum (**D**,**H**,**L**).

**Figure 2. The location and density of TMUV antigen, CD4 and CD8α molecule in the liver (LI), brain (B), spleen (SP), and small intestine (SI).** Geese were humanly killed 5 days post infection by viruses. These protein locations in the different tissues of TMUV-infected birds were detected by IHC assay. Positive virus signals were detected, cells positive for CD4 or CD8α antigen appeared dark brown using immunohistochemical staining, and sections were counterstained with haematoxylin. Rabbit polyclonal antibody against TUMV E protein was prepared by our laboratory. The dilution folds of mouse anti-duck monoclonal CD4 antibodies and mouse anti-goose monoclonal CD8α antibodies were both 1:100. Incubation of goat anti-mouse or goat anti-rabbit secondary antibody was performed by the protocols of the immunoassay kit. Liver (**A**,**E**,**I**), brain (**B**,**F**,**J**), spleen (**C**,**G**,**K**) and small intestine (**D**,**H**,**L**).

