# Supplementary material for: Antigen distribution of TMUV and GPV are coincident with the expression profiles of CD8α-positive cells and goose IFNγ
Source: Sci Rep. 2016 May 6;6:25545. doi: 10.1038/srep25545 (PMC4858762; doi:10.1038/srep25545)
Supplement: Supplementary Information [file srep25545-s1.pdf]

Antigen distribution of TMUV and GPV are coincident with the expression profiles of CD8α-positive cells and goose IFNγ

Hao Zhou[1#], Shun Chen[1,2,3,#*], Mingshu Wang[1,2,3], Renyong Jia[1,2,3], Dekang Zhu[2,3], Mafeng Liu[1], Fei Liu[3,], Qiao Yang[1,2,3], Ying Wu[1,2,3], Kunfeng Sun[1,2,3], Xiaoyue Chen[2,3], Bo Jing[3], Anchun Cheng[1,2,3*]

Supplementary data

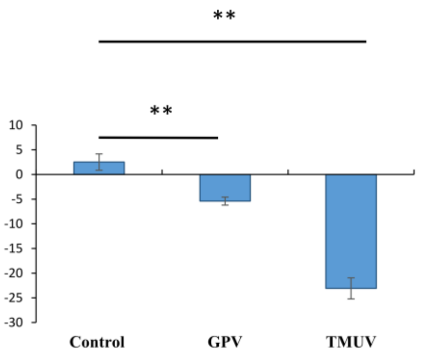

Figure. S1. The average weight change of goose groups at 5 days post infection by TMUV and GPV, respectively. Bars marked by one star (*) presented a significant difference at P<0.05; bars marked by two stars (**) presented a significant difference at P<0.01.

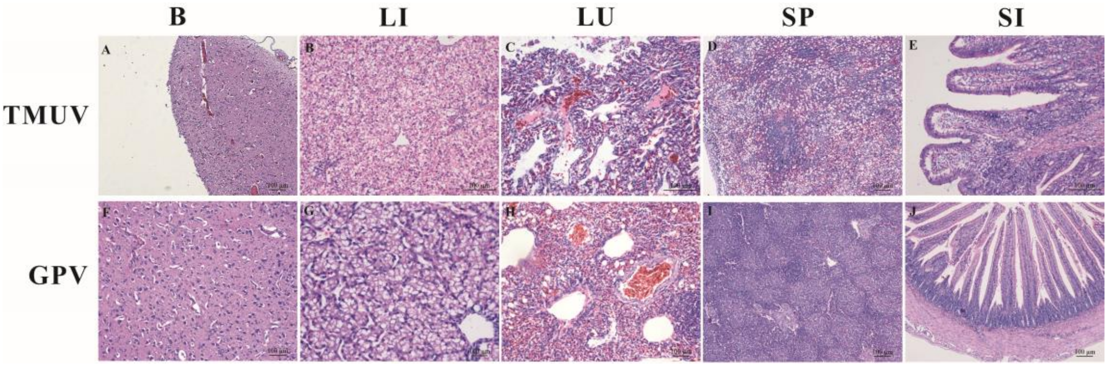

Figure. S2. Histological changes in the brain (B), liver (LI), lungs (LU), spleen (SP), and small intestine (SI) of geese infected with TMUV and GPV. The histological sections were stained with haematoxylin and eosin. Brain (A, F), liver (B, G), lungs (C, H), spleen (D, I), small intestine (E, J).
